# Cell-derived extracellular vesicles can be used as a biomarker reservoir for glioblastoma tumor subtyping

**DOI:** 10.1038/s42003-019-0560-x

**Published:** 2019-08-19

**Authors:** Rosemary Lane, Thomas Simon, Marian Vintu, Benjamin Solkin, Barbara Koch, Nicolas Stewart, Graeme Benstead-Hume, Frances M. G. Pearl, Giles Critchley, Justin Stebbing, Georgios Giamas

**Affiliations:** 1 Department of Biochemistry and Biomedicine, School of Life Sciences, University of Sussex, Brighton, BN1 9QG UK; 2 Pharmacy and Biomolecular Sciences, University of Brighton, Brighton, BN2 4GJ UK; 3 Bioinformatics Group, School of Life Sciences, University of Sussex, Falmer, Brighton, BN1 9QG UK; 4 Department of Neurosurgery, Hurstwood Park Neurosciences Centre, Brighton and Sussex University Hospitals, Brighton, UK; 5 Department of Surgery and Cancer, Division of Cancer, Imperial College London, Hammersmith Hospital Campus, Du Cane Road, London, W12 ONN UK

**Keywords:** Head and neck cancer, Tumour biomarkers

## Abstract

Glioblastoma (GBM) is one of the most aggressive solid tumors for which treatment options and biomarkers are limited. Small extracellular vesicles (sEVs) produced by both GBM and stromal cells are central in the inter-cellular communication that is taking place in the tumor bulk. As tumor sEVs are accessible in biofluids, recent reports have suggested that sEVs contain valuable biomarkers for GBM patient diagnosis and follow-up. The aim of the current study was to describe the protein content of sEVs produced by different GBM cell lines and patient-derived stem cells. Our results reveal that the content of the sEVs mirrors the phenotypic signature of the respective GBM cells, leading to the description of potential informative sEV-associated biomarkers for GBM subtyping, such as CD44. Overall, these data could assist future GBM in vitro studies and provide insights for the development of new diagnostic and therapeutic methods as well as personalized treatment strategies.

## Abstract (web summary)

Lane, Simon, Vintu et al. analyze the protein content of small extracellular vesicles produced by different glioblastoma cell lines and patient-derived stem cells. They show that the proteomic cargo of small extracellular vesicles can be used as biomarkers for glioblastoma subtyping.

## Introduction

Glioblastoma multiforme (GBM) is amongst the most aggressive types of brain tumors for which current treatments are of limited benefit^1^. Verhaak et al. has previously described different clinical genetic GBM subtypes (proneural, neural, mesenchymal, and classical) based on the gene expression of different markers, such as platelet-derived growth factor-receptor alpha (PDGF-Rα), neurofilament light (NEFL), CD44, and epidermal growth factor-receptor (EGF-R), respectively^2^. This sub-classification might have diagnostic and prognostic applications as, for example, the mesenchymal subtype is acknowledged as the most aggressive one^3,4^. Nevertheless, all these subtypes can co-exist within the same tumor, making patients’ sub-classification challenging^5^. In addition, according to recent reports, the neural subtype may simply represent normal brain contamination^6^.

During GBM growth, the close crosstalk between the different components of the integrated GBM microenvironment, including the hyaluronic acid (HA)-rich extracellular matrix and stromal cells, such as endothelial cells or astrocytes, can support tumor invasiveness and resistance to therapy^7^. In addition, an important role in tumor recurrence and resistance to treatment is attributed to GBM stem cells present in the tumor bulk as they are less affected by radio-therapy and chemo-therapy^8–10^. Such resistance is further supported by GBM stem cells capabilities to generate different GBM cell sub-populations of various molecular signatures^11,12^. Intra-tumoral heterogeneity is therefore a central feature of GBM tumors, although it has not been fully described to date^13,14^. Nevertheless, a better understanding of GBM heterogeneous sub-populations/molecular signatures would be of great help for future in-depth studies and, eventually, novel therapeutic strategies.

Extracellular vesicles (EVs) represent one of the plausible ways through which tumor cells, including cancer stem cells, self-regulate and communicate with their stromal counterparts and hence maintain such high intra-tumoral heterogeneity^15^. EVs are membrane-enclosed vesicles that can carry proteins, lipids, metabolites, and nucleic acids from one cell to another, for short or long distances^16,17^. In GBM, EVs have been described to be involved in tumor invasion, neo-angiogenesis, modulation of the immune response and resistance to treatments such as temozolomide^18–20^. Recent reports have focused on the role of small EVs (sEVs, <200 nm diameter) in cancer progression, as opposed to medium/large EVs (m/lEVs, >200 nm)^15,21^. We have recently reported that shedding of bevacizumab, an antibody neutralizing VEGF-A, at the surface of GBM cell-derived sEVs might be involved in the tumor resistance to anti-angiogenic therapies^22^. Furthermore, recent reports suggested that GBM cells of distinct subtypes/molecular signatures accordingly produce EVs with different contents^3,23,24^. Indeed, Spinelli et al. showed that proneural and mesenchymal GBM stem cells produce different EVs in terms of proteomic content and pro-angiogenic effects^23^. By describing the proteomic cargo of GBM cell line-derived EVs, Mallawaaratchy et al. identified EV biomarkers that are potentially associated with higher GBM invasiveness, such as Annexin A1 and Integrin ß1. Interestingly, through a gene expression analysis of GBM specimens, authors reported that Annexin A1 expression is higher in the mesenchymal and classical subtypes, suggesting a survival/subtype prediction potential for EV-associated Annexin A1^3^. Similarly, blood-derived and cerebrospinal fluid (CSF)-derived EV cargos have been recently proposed as good biomarker candidates for diagnosing GBM and describing specific subtypes/molecular signatures, and also for assessing tumor resistance to existing therapies^18,25,26^. Indeed, the presence of EVs in biofluids along with their capability to cross the blood brain barrier, makes them very valuable carriers of potential GBM biomarkers, while current methods for the purposes of diagnosis/prognosis are still painful and invasive^15,27,28^. Recently, Osti et al. reported higher levels of EVs in GBM patients compared to healthy controls, suggesting a new potential method to help GBM diagnosis^24^. Nevertheless, there is still a great need for identifying precise EV-associated biomarkers that could help determine specific GBM tumor subtype/molecular signatures in patients. In addition, as the EV field is constantly evolving and relies a lot on fast growing and highly EV-producing tumor cell lines, an extended description of available models for EV-related GBM research would be of great value.

For all these reasons, our aim was to describe the proteomic content of sEVs derived from GBM cells with various molecular signatures. We first grouped GBM cell lines and patient-derived stem cells according to the expression of specific key markers and their in vitro invasiveness potential. Interestingly, we were able to associate some of the cell sub-groups that we identified to GBM subtypes/molecular signatures that have already been described^2^. Ultimately, we observed that description of the proteomic content of the GBM cell-derived sEVs mirrored our original cell grouping. Consequently, this extensive study led to the identification of new potential sEV-associated protein biomarkers that can be used as indicators of GBM aggressiveness and assist in GBM subtype classification.

## Results

### In vitro invasion capabilities of astrocytes and GBM cells

**Fig. 1:**
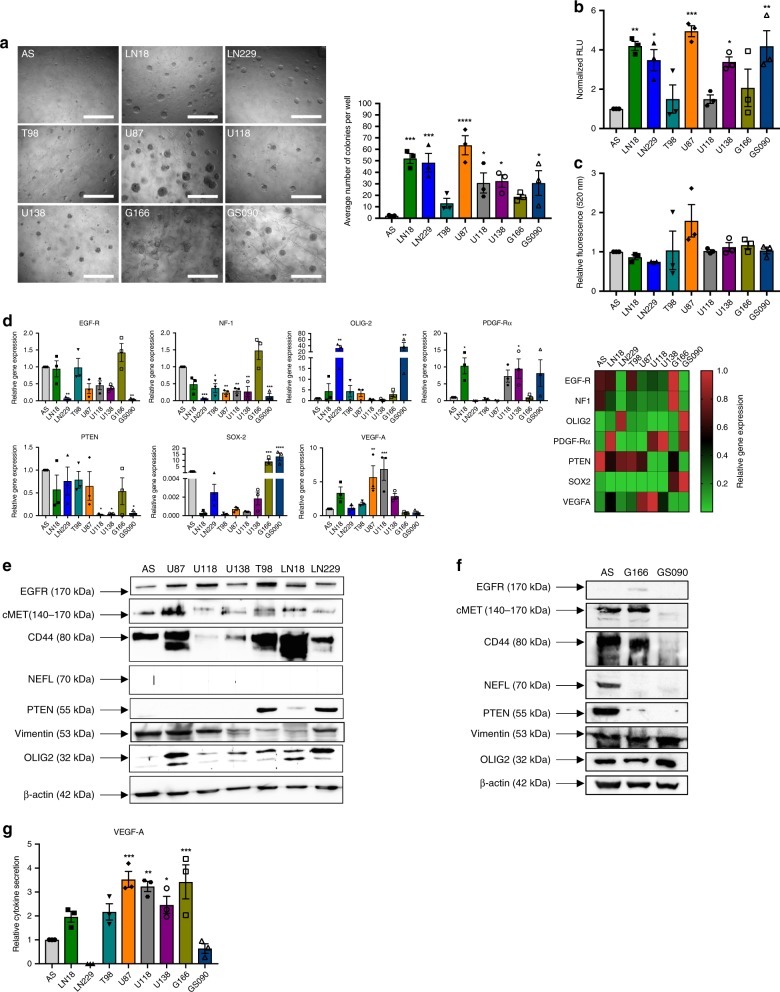
Astrocytes (AS), GBM cell lines, and GBM patient-derived stem cells present different in vitro invasion capabilities and specific subtype marker expression. **a** AS and GBM cells invasiveness and colony formation abilities using a hyaluronic acid (HA)-based hydrogel assay. Cells were incubated within a HA hydrogel for 7 days. Colony counting was then performed. Scale bar = 400 µm. **b** AS and GBM cell viability in a HA hydrogel-based assay using the CellTiter-Glo^®^ Luminescent Cell Viability Assay. **c** Invasion abilities of AS and GBM cells through an extracellular matrix-coated membrane. Cells were seeded in the top chamber and were allowed to invade the matrix for 24 h in presence or absence of FCS in the bottom chamber. Cells that have passed through the matrix were then detached, lysed, and labeled with CyQuant GR Dye. Fluorescence was then read (480/520 nm filter set). Data obtained in presence of FCS was normalized to data obtained without FCS. Representative images are shown. **d** qRT-PCR analysis of GBM subtype and aggressiveness marker expression in astrocytes, six different GBM cell lines and two different GBM patient-derived stem cells. GAPDH was used as an internal control. Data are shown as normalized to AS data. Heat-map representative of the qRT-PCR data where the data is normalized to the highest level of gene expression. **e** Western blotting analysis of GBM subtype and aggressiveness marker expression in AS and six different GBM cell lines. β-actin was used as an internal control. **f** Western blotting analysis of GBM subtype and aggressiveness marker expression in astrocytes and two different GBM patient-derived stem cells. β-actin was used as an internal control. **g** ELISA analysis of VEGF-A secretion by AS, six different GBM cell lines and two different GBM patient-derived stem cells. Representative images are shown. The mean ± SEM of *n* = 3 independent experiments is shown. *p < 0.05, **p < 0.01, ***p < 0.001, ****p < 0.0001 (ordinary one-way ANOVA)

Invasion assays in 3D HA-hydrogels were undertaken to determine the colony forming abilities of astrocytes and GBM cells (Fig. 1a and Supplementary Data 1). LN18, LN229, and U87 cells formed the highest number of colonies with an average of 52, 48, and 64 colonies per well, respectively (*p* = 0.0003, *p* = 0.0008, and *p* = 0.0001 compared to astrocytes, respectively), while U118, U138, and GS090 cells had 31, 32, 19, and 31 colonies, respectively (*p* = 0.0407, *p* = 0.0283, and *p* = 0.0417 compared to astrocytes, respectively). T98, G166 cells, and astrocytes’ number of colonies were significantly lower (13 and 2 per well, respectively, *p < 0.05, **p < 0.01, ***p < 0.001, ****p < 0.0001, ordinary one-way ANOVA) (Fig. 1a and Supplementary Data 1). To further describe the GBM cells’ behavior when growing in the HA hydrogels, we then performed a cell viability assay (Fig. 1b and Supplementary Data 1). The relative viability, expressed here in Relative Light Units (RLU), was 4.2, 3.5, 4.95, 2.4, and 4.2 fold higher in LN18, LN229, U87, U138, and GS090 cells, respectively, when compared to astrocytes (*p* = 0.0033, *p* = 0.0251, *p* = 0.0004, *p* = 0.0323, and *p* = 0.0034 compared to astrocytes, respectively). The viability of T98, U118, and G166 cells was not significantly different to the astrocytes’ (*p < 0.05, **p < 0.01, ***p < 0.001, ****p < 0.0001, ordinary one-way ANOVA) (Fig. 1b and Supplementary Data 1).

Further assays were implemented to complete our understanding of the migration, proliferation, and invasion capabilities of the studied cells. As presented in Fig. 1c, U87 cells were able to invade through a basement membrane matrix-coated insert more than any of the other cell lines. Indeed, when compared to the control (no FCS), U87 cells migrated 66% more into the matrix in the presence of FCS in the lower chamber (Fig. 1c and Supplementary Data 1). In addition, as shown in Supplementary Fig. 1A, AS, U118 and U138 showed the highest migration potential with 77%, 76%, and 72% wound healing, while LN229 and U87 cells had a 43% and 53% closure, respectively (Supplementary Data 2). LN18 and T98 cells’ wound healing abilities were significantly lower than the one observed in AS(40%, *p* = 0.0441 and 7%, *p* = 0.0003 respectively, *p < 0.05, **p < 0.01, ***p < 0.001, ****p < 0.0001, ordinary one-way ANOVA). Furthermore, U87 cells presented the shortest population doubling time (25.5 h, *p* = 0.0019 compared to AS), followed by LN229, LN18, and T98 (26.6 h, *p* = 0.0023, 28.2 h, *p* = 0.0029 and 30.1 h, *p* = 0.0039 respectively, *p < 0.05, **p < 0.01, ***p < 0.001, ****p < 0.0001, ordinary one-way ANOVA) (Supplementary Fig. 1B and Supplementary Data 2).

Taken together, our results confirm that AS are quite motile in 2D albeit their low invasiveness potential in 3D. T98 cells had equally limited migration and invasion capabilities. Despite their restricted in vitro motility, showing both low wound healing and basement membrane invasion, LN18 and LN229 GBM cells were significantly more invasive in HA hydrogels compared to AS (second and third most invasive, respectively) and had short population doubling times. U87, U118, and U138 cells presented higher migration abilities compared to the other GBM cells and significantly higher invasiveness in HA hydrogels vs. the AS. Among these, only U87 cells displayed the highest basement membrane matrix invasion along with the shortest population doubling time. Amongst the stem cells, only GS090 showed a significantly higher invasive potential, in the HA hydrogels, vs. the AS. Overall, from all the GBM cells that we analyzed, U87 cells had the highest invasion capabilities.

### Expression of signature markers in GBM cells and astrocytes

Using the Verhaak et al. classification, we then assessed in our cell line panel the expression of different markers related to the (i) ‘classical’ (EGF-R), (ii) ‘mesenchymal’ (Neurofibromatosis type 1 (NF1), CD44), (iii) ‘proneural’ (PDGF-Rα, Oligodendrocyte transcription factor 2 (OLIG2), SOX2), or (iv) ‘neuronal’ (NEFL) signatures^2^. In addition, the expression levels of PTEN, vimentin, and vascular endothelial growth factor-a (VEGFA) have been determined with the aim of obtaining further information regarding the tumor cells’ aggressiveness.

Gene expression analysis showed significantly lower levels of *EGF-R* in LN229 and GS090 cells compared to AS (95% lower, *p* = 0.005 and *p* = 0.0041 respectively), while *NF1* levels were significantly <50% in all GBM cells when compared to AS, except from G166 cells (+47% compared to AS). *OLIG-2* appeared to be expressed ~30× more in LN229 (*p* = 0.0072) and GS090 (*p* = 0.0023) cells than in AS while its levels were low in the other GBM cells. *PDGF-Rα* expression was observed at its highest in LN18 (*p* = 0.0182) and U138 (*p* = 0.0337) cells (10×-fold and 9×-fold higher compared to AS, respectively). *PTEN* was present at similar extents in most of the cells, including the AS, except from the U118 (*p* = 0.0292), U138 (*p* = 0.0323), and GS090 (*p* = 0.0368) cells where it was hardly detectable. Finally, regarding *VEGF-A*, only U87 and U118 cells showed significantly higher levels (>5×-fold higher, *p* = 0.0074 and *p* = 0.0009, respectively) vs. the AS (*p < 0.05, **p < 0.01, ***p < 0.001, ****p < 0.0001, ordinary one-way ANOVA) (Fig. 1d and Supplementary Data 3).

Most of these discrepancies were recapitulated by western blotting (Fig. 1e, f, Supplementary Figs. 2 and  3). PTEN was mainly detected in T98 and LN229 cells, while CD44 was highly expressed in LN18, T98, U87, and G166 cells. Similarly, c-Met was over-expressed in U87, T98, LN18, and G166 cells. Regarding NEFL, its expression could only be observed in AS as well as in U118 and U138 cells (Fig. 1e, f, Supplementary Figs. 2 and 3). Finally, ELISA assays demonstrated that VEGF-A cytokine secretion is significantly higher in U87 (*p* = 0.0003), U118 (*p* = 0.0011), U138 (*p* = 0.0359), and G166 (*p* = 0.0005) cells than in AS (*p < 0.05, **p < 0.01, ***p < 0.001, ****p < 0.0001, ordinary one-way ANOVA) (Fig. 1g and Supplementary Data 4).

In summary, our genomic/proteomics analyses revealed distinctive expression of GBM subtype markers within the panel of GBM and stem cells that were tested.

### Clustering of GBM cells into different signatures

**Fig. 2:**
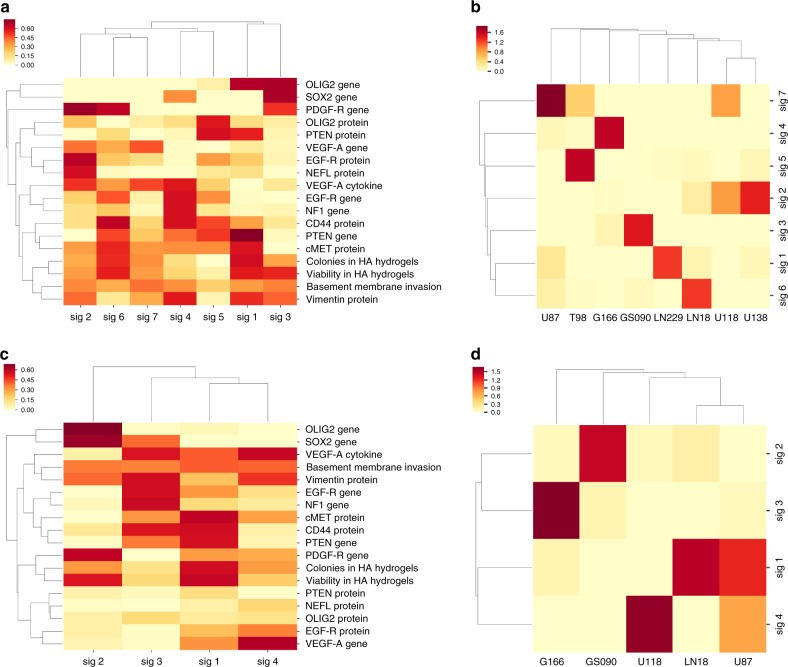
Different groups of GBM cells can be defined based on invasiveness potential and marker expression data. **a** Clustering heatmap for each parameter shown in Fig. 1, based on the phenotype and marker expression data across all cell lines. Parameters have been clustered in seven different signatures (sig 1–7) in order to reduce the dimensionality of the data. **b** Clustering heatmap for GBM cells based on the signatures defined in **a**. GBM cells have been grouped based on this correlation analysis (U87/T98/G116/GS090/LN229/LN18/U118 & U138). **c** Clustering heatmap for each parameter shown in Fig. 1, based on the phenotype and marker expression data in LN18, U87, U118, G166, and GS090. Parameters have been clustered in four different signatures (sig 1–4) in order to reduce the dimensionality of the data. **d** Clustering heatmap for LN18, U87, U118, G166, and GS090 GBM cells based on the signatures defined in **c**. GBM cells have been grouped based on this correlation analysis (G166/GS090/U118/LN18 & U87)

The invasiveness and gene/protein markers’ expression data presented in Fig. 1 were put together and compared through clustering analysis resulting in the identification of seven distinctive signatures using non-negative matrix factorization (Fig. 2a and Supplementary Data 5). Then, based on these expression signatures, GBM and stem cells have been compared and clustered together according to their similarities. As shown in Fig. 2b, U118 and U138 were grouped together in a common sub-cluster while U87, T98, G166, GS090, LN229, and LN18 failed to cluster with any other studied GBM cell line (Supplementary Data 5).

Our analysis revealed that LN18, U87, U118, G166, and GS090 GBM cells express distinct parameter/marker signatures, suggesting that they could represent distinct GBM signatures. Hence, a separate four signature clustering of the parameters shown in Fig. 1 has been generated to further describe the LN18, U87, U118, G166, and GS090 cell lines (Fig. 2c and Supplementary Data 5). Signature 1 was mostly characterized by high VEGF expression, high CD44 protein expression, high cMET protein expression, low PTEN protein expression, and high invasiveness potential (high number of ‘colonies in HA hydrogels’, ‘Viability in HA hydrogels’ and ‘Basement membrane invasion’) (Fig. 2c and Supplementary Data 5). Signature 2 was mostly characterized by high PDGF-R, OLIG2, and SOX2 gene expression, as described in GS090 GBM cells (Fig. 2d and Supplementary Data 5). Inversely, signature 3 showed high vimentin protein expression associated with high CD44 protein expression, high NF1 gene expression and high EGF-R gene expression. As seen in Fig. 2d, signature 3 was mainly observed in G166 GBM cells. Signature 4, which was observed in U118 GBM cells, was defined by high VEGF-A gene expression production as well as high vimentin protein expression (Fig. 2d and Supplementary Data 5). Finally, as shown in Fig. 2d, a strong association of signature 1 with LN18 and U87 GBM cells could be observed. In addition, cosine similarity assay confirmed the high similarity between LN18 and U87 (Supplementary Fig. 4 and Supplementary Data 6).

### Analysis of GBM cell-derived sEVs size and concentration

Based on our aforementioned clustering results, we decided to focus on these five distinct GBM cells, namely LN18, U87, U118, G166, and GS090. Description of their respective sEV production and proteomic cargo was undertaken in an attempt to identify GBM signature markers in their EVs.

**Fig. 3:**
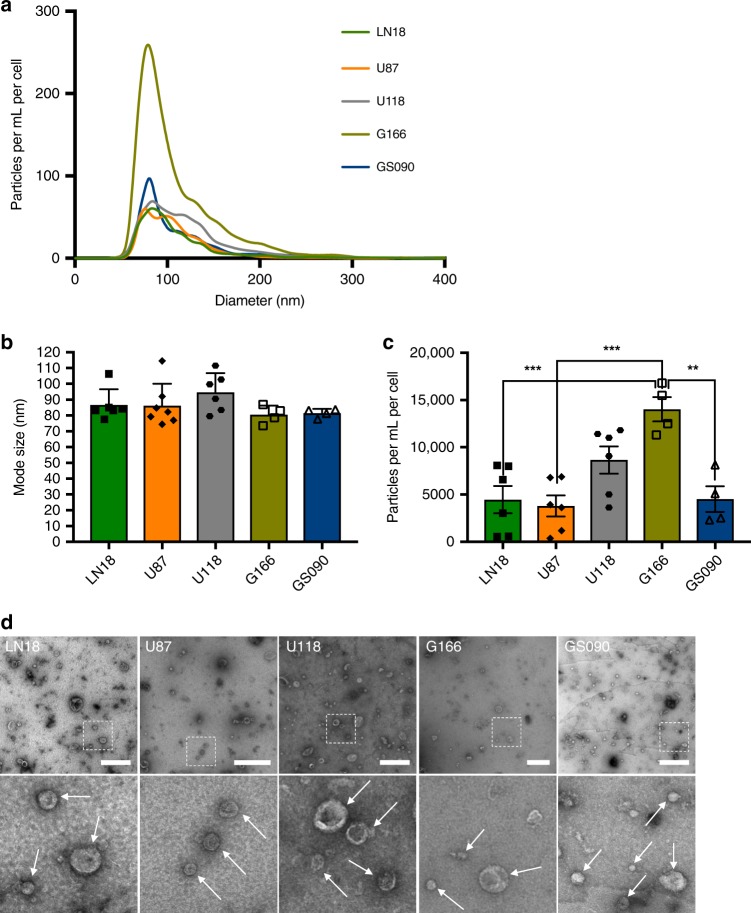
sEV fractions produced by different GBM cell lines and patient-derived stem cells show variable concentrations and specific patterns of EV markers expression. **a** NTA of GBM cell-derived sEVs. sEV suspension was 1/50 diluted and infused into a Nanosight^©^ NS300 instrument. Five captures of 60 s each were recorded. Particle concentration (particles/mL) and size (nm) were measured. Particles concentration was normalized to the number of cells (particles/mL/cell) at CM harvest. The mean of at least four independent experiments is shown. **b** Mode size (nm) distribution of GBM cell-derived sEVs. sEV mode sizes were determined by NTA. **c** Concentrations (particles/mL/cell) of GBM cell-derived sEVs. sEV concentrations were determined by NTA. **d** TEM detection of GBM cell-derived sEVs (×20k magnification and zoom). White arrows show sEVs. Representative pictures are shown. Scale bar = 500 µm. The mean ± SEM of at least *n* = 4 independent experiments is shown (LN18 *n* = 6, U87 *n* = 7, U118 *n* = 6, G166 *n* = 5, GS090 *n* = 4). *p < 0.05, **p < 0.01, ***p < 0.001, ****p < 0.0001(ordinary one-way ANOVA)

Size distribution and concentration of sEVs derived from the selected GBM cells were initially determined by NTA. As shown in Fig. 3a, EV concentration (particles /mL/cell) at the size mode was: 60.3 particles/mL/cell for LN18, 59.9 particles /mL/cell for U87, 69 particles /mL/cell for U118, 259.2 particles /mL/cell for G166 and 97.2 particles /mL/cell for GS090. The average EV size modes were: 86.6 nm for LN18, 86.3 nm for U87, 94.6 nm for U118, 80.48 nm for G166 and 81.5 nm for GS090 (Fig. 3b and Supplementary Data 5). Total sEV concentration was 4460 particles /mL/cell for LN18, 3790 particles /mL/cell for U87, 8650 particles /mL/cell for U118, 14,000 particles /mL/cell for G166 and 4520 particles /mL/cell for GS090 GBM cells. As shown in Fig. 3c, concentration of sEVs produced by G166 GBM stem cells was significantly higher than the concentration of sEVs produced by either LN18 (*p* = 0.0009), U87 (*p* = 0.0004) or GS090 (*p* = 0.0025) GBM cells (*p < 0.05, **p < 0.01, ***p < 0.001, ****p < 0.0001, ordinary one-way ANOVA) (Fig. 3c and Supplementary Data 7).

Furthermore, coupled to the NTA results, the TEM pictures in Fig. 3d showing vesicles in the well-described size range of 50–150 nm further confirmed the EV isolation from the different GBM cell culture CM^17^.

### Mass spectrometry (MS) analysis of GBM cell-derived sEVs

**Fig. 4:**
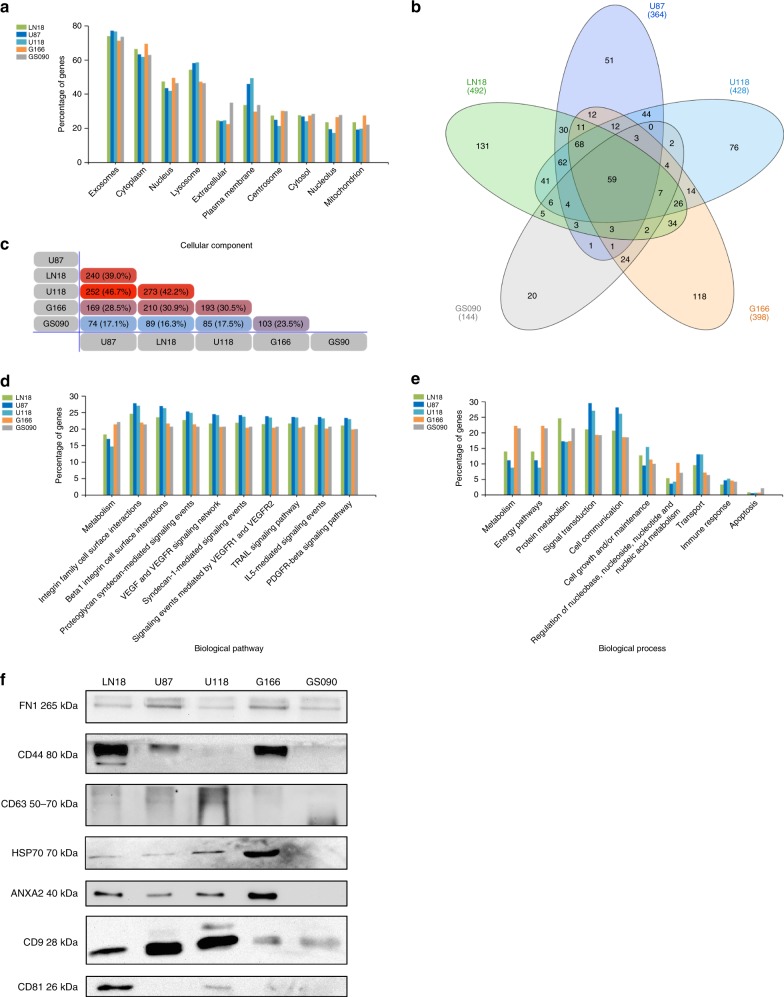
MS analysis reveals sEV proteomic content that mirrors GBM cell clustering signature and invasiveness in vitro. Protein hits were identified in GBM cell-derived sEVs via MS. Only the protein hits common to at least three biological repeats were considered for each cell line/stem cell (LN18 *n* = 3, U87 *n* = 3, U118 *n* = 4, G166 *n* = 5, GS090 *n* = 4). **a** Gene enrichment analysis for ‘Cellular component’ was performed based on the MS hits identified from each GBM cell-derived sEVs. **b** Venn diagram based on the identified MS hits. **c** Pairwise comparison diagram showing similarity between the proteome contents of the different GBM cell-derived sEVs. **d** Gene enrichment analysis for ‘Biological pathway’ was performed based on the MS hits identified from each GBM cell-derived sEVs. **e** Gene enrichment analysis for ‘Biological process’ was performed based on the MS hits identified from each GBM cell-derived sEVs. **f** Western blotting detection of fibronectin (FBN), CD44, CD63, HSP70, AnnexinA2 (ANXA2), CD9, and CD81 in GBM cell-derived sEVs

Using MS, the proteomic content of the sEVs derived from LN18, U87, U118, G166, and GS090 has been deciphered (Fig. 4 and Supplementary Data 8). Gene enrichment analysis for ‘Cellular component’ confirmed the ‘exosomes’ origin of most of the identified proteins (>70% of genes in all GBM cell-derived sEVs) (Fig. 4a). Venn diagrams (Fig. 4b, c) revealed the maximum protein expression overlap between U118 and U87 GBM cell-derived sEV content (46.7%). Proteomic content of sEVs derived from LN18 GBM cells mostly overlapped with the content of U118 (42.2%) and U87 (39.0%) GBM cell-derived sEVs. Proteomic content of sEVs derived from GS090 showed low similarity (<25%) to any other GBM cell-derived sEV proteomic content, with the highest overlap observed with the G166 GBM cell-derived sEV content (23.5%). Altogether, as shown in Fig. 4c, grouping of GBM cell-derived sEV proteomic content distinctly clustered LN18, U87, and U118 together as opposed to G166 and GS090 GBM cells (Fig. 4 and Supplementary Data 8).

Similarly, gene enrichment analysis for ‘Biological pathways’ and ‘Biological processes’ showed enrichment of the ‘Metabolism’ pathway and Metabolism’ and ‘Energy pathways’ processes in the proteomic content of sEVs derived from G166 and GS090 GBM cells, as opposed to the other tumor cell-derived sEVs (Fig. 4d, e). Inversely, our analysis revealed enrichment of pathways such as ‘Beta1 integrin cell surface interactions’, ‘Proteoglycan syndecan-mediated signaling events’ or ‘VEGF and VEGFR signaling network’ in the proteomes of sEVs derived from LN18, U87, and U118 GBM cells. Processes such as ‘Signal transduction’ and ‘Cell communication’ were also predominant (>25%) in sEVs derived from those GBM cells (Fig. 4d, e, Supplementary Fig. 5 and Supplementary Data 8).

A further detailed analysis of the proteomes of the studied GBM cell-derived sEVs showed a shared expression of known EV markers or proteins commonly present in EVs, such as Annexin A2 (ANXA2), CD63, fibronectin (FN1), GAPDH, or tubulin (TUBB). Furthermore, other EV markers such as CD82, CD81, CD9, TSG101, or ADAM10 could be detected in sEVs derived from LN18, U87, and U118. CD82 was also observed in G166 GBM cell-derived sEVs (Fig. 4 and Supplementary Data 8). Notably, CD44, a now well-described marker of aggressive mesenchymal GBM, was identified in sEVs derived from LN18, U87, U118, and G166 GBM cells^29^ (Supplementary Fig. 6 and Supplementary Data 9).

As we initially grouped LN18 and U87 GBM cells together (signature 1) showing the highest levels of in vitro invasiveness (Fig. 2), we thoroughly looked for potential relevant markers of GBM aggressiveness among the protein hits exclusively present in both sEV fractions derived from these cell lines. By doing so, we identified WNT5a, TGFBI, and SERPINE1, all recently associated with the GBM mesenchymal subtype and tumor invasion^30–32^, as well as GDF-15, also known to be linked to GBM progression and poor prognosis^33,34^. TCGA data confirmed the significant association of a high expression of SERPINE1 and TGFBI with mesenchymal subtype in GBM patients (Supplementary Fig. 6 and Supplementary Data 9).

Finally, we further evaluated the distribution of specific markers in sEVs produced by the different GBM cells, including fibronectin, CD63, HSP70, Annexin A2, CD9 CD81, as well as CD44 which has been recently observed at the surface of EVs from different sources, such as ovarian and breast cancer cells but also mesenchymal stem cells, whilst being associated with GBM progression and aggressiveness^23,29,35–37^.

Overall, Fig. 4f show that sEVs derived from signature 1-associated LN18 and U87 GBM cells display similar levels of CD63, HSP70, and Annexin A2 while sharing the highest expression of CD63 and CD9 with signature 4 (U118 cells), as compared to sEVs derived from G166 and GS090 stem cells. Both sEV fractions from G166 and GS090 cells had low levels of CD63 and CD9 expression. Highest expression of FBN was observed in U87 and G166 GBM cell-derived sEVs. Furthermore, CD44 was clearly detected only in LN18, U87, and G166 GBM cell-derived sEVs (Fig. 4f and Supplementary Fig. 7).

## Discussion

Distinct molecular subtypes have been defined in order to make GBM diagnosis more precise, with direct links to tumor aggressiveness and patients overall survival^2^. Nevertheless, clinical application of such subtyping is still quite limited, due to a lack of reliable and accessible biomarkers. For these reasons, the present study aimed to describe markers for specific GBM signatures in sEVs derived from tumor cells, according to their in vitro invasion potential. We believe that such biomarkers should be detectable in sEVs derived from patients’ biofluids (i.e. blood or CSF), thus helping diagnosis and development of future personalized therapies^3^.

Indeed, by correlation clustering of our phenotypic and molecular results, we could define distinct signatures to describe the GBM cells that were employed in this study. Interestingly, in accordance with the widely used Verhaak classification and other recent reports, signatures 1 and 2 presented characteristics specifically associated with the mesenchymal and proneural subtype, respectively. Indeed, as often reported for the mesenchymal GBM subtype, signature 1 was mostly characterized by high CD44 and cMET expression, as well as high cell invasiveness. Similarly, proneural markers such as high PDGF-R and OLIG2 expression were the main parameters linked to signature 2. Signature 3 and 4 could not be clearly linked to any of the described GBM subtypes even though signature 3 presented the highest EGF-R gene expression, a marker for the classical GBM subtype.

Interestingly, the cell clustering was mirrored in the proteomic content of sEVs derived from these GBM cells. Indeed, according to our MS data and gene-enrichment analysis, there was a clear separation between LN18, U87, and U118 on one side and G166 and GS090 on the other. Such discrepancy was further supported by the identification of biological pathways and processes in the sEV proteomes. According to our analysis, the content of EVs derived from U87, U118, and LN18 appeared similar while being enriched in signaling pathways, such as ‘Integrin family cell surface interaction’ or ‘VEGF and VEGFR network’, known to be directly linked to GBM progression. On the other hand, the EV proteomic signature of G166 and GS090 cells was mostly related to ‘normal conditions’ machinery/metabolism pathways, e.g. ‘Energy pathways’ or ‘Metabolism’^38,39^. Such discrepancies in the EV cargo between GBM cell lines and GBM stem cells may be due to the remarkable metabolic flexibility of cancer stem cells, as opposed to normal/proliferative cancer cells^40,41^, which can have a direct impact on the EV cargo of GBM stem cells^15^.

Moreover, differences could also be observed when looking closely at the expression levels of sEV specific markers, such as CD63, HSP70, Annexin A2, and CD9. sEVs derived from LN18, U87, and U118 GBM cells had similar expression patterns when compared to GBM stem cell-derived sEVs. Furthermore, sEVs from signature 2 (GS090 stem cells) only showed clear expression of CD9 and fibronectin, as recently reported in a similar way for ‘proneural’ GBM cell-derived EVs by Spinelli et al.^23^. In accordance with our present data, authors indeed showed that the GBM stem cell subtype affected EV molecular characteristics as sEVs produced by proneural GBM stem cells had very low levels of CD9, CD63, and CD81 expression compared to sEVs derived from mesenchymal GBM stem cell cultures^23,42^. Finally, the similarities spotted between G166 and LN18 cells (signature 1) and between U118 and U87 cells (signature 4) were partially recapitulated in our proteomic analysis of the EVs-content. Such nuances could be related to the differences in the in vitro migration/invasion capabilities we observed between LN18 and U87 GBM cells. As both cell lines show mesenchymal features (signature 1), we consider LN18 cells to be in an intermediate mesenchymal state, as opposed to the fully invasive mesenchymal U87 cells. Indeed, one could argue that such observation appears similar to a ‘go-or-grow’ model where LN18 cells would rather ‘grow’ into a ‘tumor friendly’ microenvironment characterized by HA abundance than ‘go’ and migrate through the basement membrane and further invade surrounding tissue layers^43,44^.

Taken together, based on the current MS data and specific sEV markers expression, LN18, U87, and U118 appeared to cluster together, as similarly observed in our four-signature clustering that grouped signature 1 (LN18 and U87) along with signature 4 (U118), while our 7-signature clustering also initially grouped LN18 and U118 GBM cells together. Yet, LN18 and U87 on one hand and U118 on the other hand clearly differ in terms of cMET and CD44 expression, as well asin their invasiveness in the HA hydrogels^45^. As a matter of fact, the low expression of CD44 in sEVs derived from U118 GBM cells seems indicative of such difference with LN18 and U87 cells. Our data suggest that sEV-associated CD44 expression could be correlated with GBM cell invasiveness. Yet, as high CD44 has been detected in sEVs derived from low invasive G166 cells, and in accordance with the rest of our results, we thus think that distinct GBM signatures/subtypes might be differently associated with exclusive expression levels of a few selected EV-associated markers. Accordingly, recent reports suggested that profiling the expression of surface EV proteins could provide cancer diagnostic signatures from biofluids^36^.

Along with CD44, our results suggest TGFBI and SERPINE1 (PAI-1) as potential sEV-associated biomarkers for the aggressive mesenchymal subtype^31,32^. We especially focused on CD44 as it has often been associated with the mesenchymal phenotype and cell invasion in GBM^29,37,45^. Accordingly, EV-associated CD44 has been linked to tumor progression and resistance to treatment in breast cancer and myeloma, respectively^37,46,47^. Furthermore, we suggest that such biomarkers could help the follow-up of GBM tumors and the monitoring of recurrence/treatment resistance^24^. In the same way, an increase of the expression of proteins, such as ECM1, CD9, and CD44 has been reported in EVs derived from squamous cell carcinoma cells upon mesenchymal transformation^48,49^. Altogether, both our data and recent publications suggest that changes in the EV-specific marker expression patterns could help identify highly invasive/aggressive tumors.

A few studies have already reported EV-associated markers that could be used for discriminating GBM from normal and stromal CNS cells, such as annexins and integrins^3,42^. Combined with deciphering the expression of specific EV markers and EV-associated GBM subtype markers, such integrated approaches should provide an accurate diagnosis with potential subtype characterization. Nevertheless, both cellular and molecular heterogeneity has been repeatedly reported in GBM tumors^2^. For these reasons, characterizing a GBM tumor subtype based on the respective EV proteomic content appears quite challenging, as markers from various subtypes might be present in patients’ samples^5^. Nevertheless, precise quantification of the EV-associated markers should give further information regarding the tumor main molecular signature and, consequently, associated prognosis.

We believe that this study supports the clinical potential of the content of EVs derived from different GBM subtypes^26^. According to our data, EVs may contain reliable protein markers, in particular for the aggressive mesenchymal GBM subtype. Interestingly, although all the different GBM subtypes can be present in the same tumor, it has been suggested that the mesenchymal subtype takes over upon recurrence^50^. Hence, deciphering how specific GBM subtyping influences the EV cargo may help us understand how GBM can progress and recur. In the same way, patient follow-up could also benefit from such work. A limitation of the present study is the use of immortalized tumor cell lines for studying GBM subtypes, despite the concomitant use of two populations of GBM patient-derived stem cells^2^. Nevertheless, we believe that our present report can be of great help for future functional in vitro studies deciphering the role of EVs in GBM^51,52^. Yet, additional work is needed to validate our current conclusions in an in vivo setting, considering the role of the surrounding microenvironment. Furthermore, as presented by Rennert et al. RNA that is detectable in GBM EVs is a rather appealing source of biomarkers as only a small amount of genetic material is needed to perform the analysis of a few key genes. Similarly to the present study, authors suggested that describing EV content expression patterns of the four different GBM subtypes is urgently needed^26^. Also, larger vesicles, such as m/lEVs and oncosomes, might also provide meaningful information for GBM diagnosis and prognosis^21^. Finally, future translational clinical research should be performed in order to assess the application of such observations into a liquid biopsy setup^53^.

In summary, our study improves the understanding of the correlation between distinct GBM subtypes and associated potential aggressiveness with respective EV production and content. In addition, our findings suggest the existence of EV-associated biomarker patterns for GBM subtype identification in patients. Consequently, we believe that further clinical work and validation would bring new insight towards the development of more effective therapeutic strategies and personalized treatments.

## Methods

### Cells and reagents

LN18, LN229, and U118 GBM cells (ATCC) were maintained in Dulbecco’s modified Eagle medium (DMEM, Sigma-Aldrich) and T98, U87, and U138 GBM cells (ATCC) were maintained in minimum essential medium (MEM, Sigma-Aldrich). Astrocytes (Human Astrocytes, Sciencell) were maintained in Astrocyte growth medium (ASGM, Cell Applications). Poly-l-lysine (Sigma-Aldrich) at 2 µg cm^−2^ was used to coat every plastic vessel needed for astrocyte culture. Cell line culture medium was supplemented with 100 Units mL^−1^ penicillin, 100 µg mL^−1^ streptomycin, 2 mM l-glutamine (PSG, Sigma-Aldrich) and 10% heat inactivated fetal bovine serum (FBS, First Link).

G166 and GS090 (GBM patient-derived stem cells) were a kind gift from Dr. Angela Bentivegna, University of Milan-Bicocca and Dr. David Nathanson, University of California, Los Angeles, respectively. GBM stem cells were isolated from GBM tumor samples following local Ethical Board approval^54,55^. GBM stem cells were maintained as neurospheres in (DMEM/F-12, Sigma-Aldrich) completed with B-27 without Vitamin A (Life Technologies), Hu EGF (20 µg mL^−1^), Hu FGF-b (8 µg mL^−1^), Heparin (2 mg mL^−1^), 100 Units mL^−1^ penicillin, 100 µg mL^−1^ streptomycin, and Glutamax (Invitrogen). Cells were incubated at 37 °C in a humidified atmosphere at 5% CO_2_. Medium was changed twice a week. GBM cells (cell lines) and astrocytes were detached at confluence using trypsin/EDTA. GBM stem cells were disassociated using TrypleE Express Enzyme (Gibco) and separated into single cells through a 70 µm cell strainer. The International Cell Line Authentication Committee identifies U118 as a derivative of U138 as they appeared to share a common donor^56,57^. Nevertheless, considering the GBM intra-tumoral heterogeneity, we decided to use both cell lines in the present study to compare them with each other and with the rest of the cells we used^5^. All the cells were tested negative for mycoplasma at the beginning of the study.

### Cell invasion assay in HA hydrogels

Cells were incubated with HA hydrogels for 7 days according to the manufacturer’s instructions (Biomymesis, Celenys)^58^. 100,000 cells were seeded per well. All the steps conferring properties to HA hydrogels used in cell culture have been described in two Europeans patents: “Improved Crosslinked Hyaluronan Hydrogels for 3D Cell Culture” EP10305666.9, June 22, 2010 and “Method for Harvesting Cells Cultured in 3D Hydrogel Matrices” EP 10305667.7, June 22, 2010. Hyaluronan hydrogels consist of hyaluronan cross-linked with adipic dihydrazide (ADH; Sigma-Aldrich, France) and 1-ethyl-3 [3-(dimethylamino)-propyl] carbodiimide (EDCI; Sigma-Aldrich). High molecular weight hyaluronan (>106 Da; Sigma-Aldrich) is used to prepare the hydrogel plates, as originally described by Prestwich et al.^59^. Briefly, the ratios ADH:hyaluronan (10:1) and hyaluronan:EDCI (1:1) have been optimized for cell adhesion and culture. Hyaluronan and ADH are dissolved in milliQ-water. 0.1 N HCl is used to adjust the pH to 4.6. The reaction mixture is then completed with carbodiimide reagent (EDCI) and allowed to set for 2 h, with gentle agitation. Hyaluronan hydrogels are then dialyzed against 0.1 N NaCl for 2 days, then in a water:ethanol mixture (3:1 v/v) for other 2 days, and in milliQwater for 2 days in order to remove excess of ADH and EDCI. In the next step, the dialyzed hydrogel is placed in a plastic container and frozen. Following freezing, the hydrogels are placed in a lyophilizer (Alpha 1–2, Christ, Germany; performances, 2 kg ice per 24 h, *T* = −55 °C) for 4–5 days. The lyophilized hydrogels are then stored at −20 °C. Hydrogels are sterilized at 100 °C. The gel pH post-rehydration has been shown to be ~8.4. The swelling ratio of hyaluronan hydrogels at room temperature in culture medium should be 37 g g^−1^ ^60^. Colony counting was performed on six pictures randomly taken from each gel using an EVOS FLC imaging system (Life Technologies) at ×10 magnification. Cell viability was assessed using the CellTiter-Glo^®^ Luminescent Cell Viability Assay. To do so, 100 µL of Cell-Titer-Glo^®^ Reagent (CellTiter-Glo^®^ Luminescent Cell Viability Assay, Promega) was added to each well. Plate was agitated on a plate mixer for 2 min and left for 10 min at room temperature before luminescence was recorded using a GloMax Explorer plate reader (Promega). Graphs show an average of three experiments.

### Cell invasion assay through a basement membrane matrix

The QCM™ 96-well plate (Merck) was used to perform the cell invasion assay. The assay allows for measurement of cell invasion through a reconstituted basement membrane matrix. Cells were starved in serum-free medium for 24 h before the assay, according to the manufacturer’s instruction. The basement membrane matrix was rehydrated with warm cell culture medium for 2 h. Medium was discarded from the inserts and either serum-free medium (control) or 10% FCS medium was added to the feeder tray (lower chamber). 100,000 cells per well were then seeded and allowed to invade the matrix for 24 h. Following incubation, the cell suspension was carefully removed from the top chamber and inserts were rinsed in sterile PBS for 1 min. Cell detachment solution was then added to a new feeder tray and the plate was incubated for 30 min so invading cells are dissociated from underside. In order to label the cells, CyQuant GR Dye/4x Lysis Buffer solution was then added to the wells. The plate was incubated for an additional 15 min at room temperature. Finally, fluorescence was read using a GloMax Explorer plate reader (480/520 nm filter set, Promega). Data obtained in presence of FCS was normalized to data obtained without FCS. Graph shows an average of three experiments.

### Cell proliferation assay

Cells were plated in a 96-well plate (5000 cells per well) in 10% FCS medium. Cells were incubated for 24 and 96 h, washed once with sterile PBS and then fixed using 4% paraformaldehyde for 20 min. Following, cells were washed with PBS again and stained using a 0.1% crystal violet solution for 30 min. Crystal violet dye was then extracted from the cells using 10% acetic acid. Plates were placed on a plate shaker for 30 min. Absorbance was then read at 590 nm for both t24 and t96 time points using a GloMax Explorer plate reader (Promega). Doubling time (h) was calculated using this formula: doubling time = 72/(log(absorbance_590nm_ at t96) – log(absorbance_590nm_ at t24))/log2). Graph shows an average of three experiments.

### Wound healing assay

Cells were plated at 100,000 cells per well in a 24-well plate in 10% FCS medium until they reach 80% of confluence. Then, cells were washed once with sterile PBS and medium was changed for 0% FCS medium before a scratch was performed in the cell layer using a 200 µL tip. Cells were incubated for 48 h. A total of three pictures per wound were taken using an EVOS FLC imaging system (Life Technologies) at ×10 magnification. The size of the wound was then measured on each picture. Graphs show an average of three experiments. The wound healing assay could not be performed using G166 or GS090, as previously reported^61^.

### Western blotting

Cell protein lysates were extracted using RIPA buffer (Sigma) including fresh protease and phosphatase inhibitors and standard western blotting protocol was performed as described before^62^. For the EV marker analysis, 1 × 10^11^ sEVs mL^−1^ was loaded on the SDS gel. Primary antibodies: Anti-AnnexinA2 (Genscript A01471, 1/1000 dilution), anti-β-Actin (Abcam ab6276, 1/5000 dilution), anti-CD-9 (System Biosciences EXOAB-CD9A-1, 1/10000 dilution), anti-CD44 (Cell Signaling #3570, 1/1000 dilution), anti-CD63 (System Biosciences EXOAB-CD63A-1, 1/10,000 dilution), anti-CD81 (System Biosciences EXOAB-CD81A-1, 1/10,000 dilution), anti-Fibronectin (Abcam ab2413, 1/1000 dilution), anti-HSP-70 (System Bisociences EXOAB-HSP70A-1,1/10,000 dilution), anti-NEFL (Cell Signaling #2837, 1/1000 dilution), anti-OLIG2 (Genscript A01474, 1/1000 dilution), and anti-PTEN (Cell Signaling #9188, 1/1000 dilution). Secondary antibodies used: Polyclonal Goat Anti-Rabbit/Mouse Immunoglobulins/HRP (Dako P0447/8, 1/3000 dilution) antibodies and Anti-Rabbit Immunoglobulins/HRP (ExoAb antibody Kit, System Biosciences EXO-AB-HRP, 1/3000 dilution). Chemiluminescence was observed using a UVP Chemstudio instrument (Analytik Jena) and the Vision Works software. All experiments have been repeated at least three times.

### Real-time polymerase chain reaction

RNA was purified from cell pellets using the RNeasy^®^ mini kit (Qiagen) quick start protocol. Reverse transcription was carried out using a cDNA synthesis kit (Applied Biosystems). Taqman (Applied Biosystems) and cDNA were mixed with primers (Applied Biosystems) specific for the markers of interest and run on a One-Step^®^ Plus machine (Applied Biosystems). Data was evaluated using One-Step^®^ Plus software (Applied Biosystems). Each result has been normalized to GAPDH values. Graphs show an average of at least three experiments. All the primers (Table 1) were obtained from Applied Biosystems (Thermofisher), except primers for PDGF-Rα and GAPDH (Qiagen).

**Table 1:** Primers used for RT-PCR assays

Gene	Primer	Source
EGF-R	Hs01076090_m1	Applied Biosystems (Thermofisher)
PTEN	Hs02621230_s1	Applied Biosystems (Thermofisher)
NF1	Hs01035108_m1	Applied Biosystems (Thermofisher)
VEGF-A	Hs00900055_m1	Applied Biosystems (Thermofisher)
OLIG2	Hs00300164_s1	Applied Biosystems (Thermofisher)
SOX2	Hs00415716	Applied Biosystems (Thermofisher)
GAPDH	Hs99999905_m1	Applied Biosystems (Thermofisher)
PDGF-Rα	Hs_PDGFRA_1_SG	Qiagen
GAPDH	Hs_GAPDH_2_SG	Qiagen

### VEGF-A ELISA

Human VEGF DUOSET ELISA (R&D System) was used to measure VEGF-A levels in culture medium according to the manufacturer’s instructions. Absorbance was measured at 450 nm using a GloMax Explorer plate reader (Promega). Graphs show an average of three experiments.

### Extracellular vesicle concentration

In order to collect sEVs derived from GBM cell lines (LN18, U87, and U118), cells were seeded in 4–5 × 175 cm^2^ flasks and grown in 10% FCS medium until they reach confluence. Then, cells were washed with sterile PBS and 15 mL of corresponding serum-free medium was added to each flask for 24 h. Following this incubation, conditioned medium (CM) was collected and kept at either 4 °C for a very short time (up to 24 h) or at −20 °C for longer periods (up to 6 months) before sEV concentration.

To collect sEVs from GBM stem cells (G166 and GS090) in suspension cell culture (neurospheres), medium was changed at confluence (neurospheres of 150–200 µm diameter) and incubated for 24 h before CM collection. To do so, culture supernatant and neurospheres were centrifuged at 400 × *g* for 4 min and CM was collected (35 mL). CM from GBM stem cell cultures was then kept at either 4 °C for a very short time (up to 24 h) or at −20 °C for longer periods (up to 6 months) before sEV concentration. In accordance with the latest minimal information for studies of EVs, cell count at time of collection was recorded and used to normalize the final sEV concentration (particles/mL/cell)^21^.

Concentration of sEVs was performed using an ultracentrifugation-based protocol^63^. Every step of the concentration protocol was performed at 4 °C. An initial 300 × *g* centrifugation was performed for 10 min to discard any floating cells from the CM, followed by a 10 min centrifugation step at 2000 × *g* to remove any floating cell debris and dead cells (Hettich Universal 320R centrifuge). A 10,000 × *g* ultracentrifugation step (Beckman optima LE 80-k ultracentrifuge, Beckman Type 70 Ti rotor, Beckman polypropylene centrifuge 14 × 89 mm tubes, full dynamic braking, *k*_adj_ = 15,638) was then performed for 30 min to remove any further cell debris and large vesicles (m/lEVs) from the CM. Finally, a first 100,000 × *g* ultracentrifugation run was performed for 1 h30 min to pellet the sEVs (‘exosomes’) from the CM (Beckman optima LE 80-k ultracentrifuge, Beckman Type 70 Ti rotor, Beckman polypropylene centrifuge 14 × 89 mm tubes, full dynamic braking, *k*_adj_ = 494). Supernatant was stored at −20 °C. The sEV pellet was then washed in filtered sterile PBS and centrifuged again for 1 h30 min at 100,000 × *g* in order to discard contaminants. The final sEV pellet was re-suspended in 100 µL filtered sterile PBS and immediately characterized through nanoparticle tracking analysis (NTA).

Further characterization of the sEVs was performed through western blotting (see subsection ‘Western blotting’ in “Methods” section) by measuring the expression of EV membrane associated markers, such as CD63, CD9, CD81 (mainly associated with light sEVs) and fibronectin (mainly associated with dense sEVs), and EV cytosolic markers such as HSP70 and Annexin A2^17,21^.

### Nanoparticles tracking analysis (NTA)

Vesicle concentration and size were determined using a Nanosight^©^ NS300 and the Nanosight^©^ NTA 3.2 software (Malvern Instruments). The following conditions were applied for the NTA analysis at the Nanosight instrument: temperature was 20–25 °C; viscosity was ~0.98 cP; camera type was sCMOS; laser type was Blue488; camera levels were either 14 or 15; syringe Pump Speed was set to 70 AU; five measurements of 60 s each were recorded. Graphs show an average of at least four experiments.

### Transmission electron microscopy

Transmission electron microscopy (TEM) has been performed on sEV preparations in order to visualize and assess/confirm the size range of the vesicles, as described before^63^. Samples were visualized using a JEOL JEM1400-Plus (120 kV, LaB6) microscope equipped with a Gatan OneView 4K camera at ×20k magnification. 10–15 pictures per grid were taken.

### Mass spectrometry

In order to elucidate the protein content of the GBM cell-derived sEVs, MS analysis was performed. To do so, a Bradford assay was performed to determine the protein concentration of each sEV sample and 100 ng was then loaded on a SDS–PAGE gel for protein separation. Following Coomassie blue staining, 5 slices/lane were then cut out of the gel and further processed for in-gel trypsin digestion and MS run. De-staining was performed through 3 changes/washes with 50% acetonitrile (MeCN), 25 mM NH_4_HCO_3_, with 5 min shaking between each change. Reduction and alkylation were performed, respectively, with 10 mM dithiothreitol (DTT), in 25 mM NH_4_HCO_3_ (45 min at 50 °C) and 50 mM chloracetamide, 25 mM NH_4_HCO_3_ (45 min in the dark at room temperature). Subsequently, 12.5 ng µL^−1^ trypsin (in 25 mM NH_4_HCO_3_) was added to the samples, followed by an overnight incubation at 37 °C. The digest solution was then transferred to clean tubes. Next, 70% acetonitrile/5% trifluoroacetic acid was added to the gel pieces. Following 5 min shaking, the supernatant was transferred to the corresponding clean tubes. A similar further extraction was repeated another two times in order to completely dehydrate the gel pieces and consequently recover the rest of the peptides. Sample volume was reduced to 20 µL using a vacuum concentrator. Samples were then processed through a LTQ-Orbitrap mass spectrometer coupled to a Dionex NCP-3200 nanoLC system. The raw data was searched using Maxquant (Max Planck Institute of Biochemistry) against a SwissProt database (Proteome ID: UP000005640, Taxonomy: 9606—*Homo sapiens*). The following settings were used: trypsin was the enzyme with up to two missed cleavages, oxidation (M) and acetyl (Protein N-term) were set as variable modifications, Carbamidomethyl (C) was set as fixed modification, minimum peptide length was seven amino acids, maximum peptide mass was 4600 Da, minimum and maximum peptide length for unspecific search was 8 and 25 amino acids, respectively, peptides and protein false discovery rates (FDR) were both 0.01 and minimum razor + unique peptides was set to 1 (minimum of 1 peptide for protein identification). Finally, results (‘protein groups’) were exported to Microsoft Office Excel and further processed. The MS analysis was repeated at least three times for each GBM cell lines/stem lines, using independent samples. Through comparison of independent experiments for each cell line/stem cell, we described as ‘hits’ the identified proteins that appeared in at least two independent identifications. Obvious contaminants (keratins) were removed from the protein group lists. In addition, proteomic data were further deciphered by loading the gene symbols identified from the MS data in Functional Enrichment Analysis Tool (FunRich) for gene-enrichment analysis of ‘Biological pathways’, ‘Biological process’, ‘Cellular component’ and ‘Pairwise comparison diagram’. The InteractiVenn (www.interactivenn.net) online software was used to make Venn diagrams^64^.

### TCGA data

Information about the distribution of specific gene hits among the different GBM subtypes has been obtained from The Cancer Genome Atlas (TCGA) through the ‘*Expression box plot* (*Affymetrix HT HG U133A*)’ and ‘*Expression box plot (Affymetrix Human Exon 1.0 ST)*’ graphs on the Betastasis website (www.betastasis.com) that organize patients’ samples according to their GBM subtypes.

### Experimental design and statistics

Sample size was set to a minimum of three independent experiments (biological repeats) based on the magnitude and consistency of differences between cells/conditions. Experimental findings were reliably reproduced. All the results were normalized to control and reported as mean ± standard error of the mean (SEM). Ordinary one-way ANOVA tests were employed to determine the significance of the observed differences. Tukey’s test was used for multiple comparison. Differences were considered statistically significant at *p* < 0.05 (95% confidence interval, *p < 0.05; **p < 0.01; ***p < 0.001). Pearson correlation coefficient was used to measure the relationship between the considered parameters shown in Fig. 1. Mean phenotype parameter measurement across all available cell lines was decomposed into seven different signatures (sig 1–7, Fig. 2a) to reduce the dimensionality of the data and provide a method of clustering the cell lines by phenotype similarity. Additionally the LN18, U87, U118, G166, and GS090 cell lines alone were decomposed into four signatures (sig 1–4, Fig. 2c). This decomposition was achieved using non-negative matrix factorization (NMF). Each cell-line’s mean parameter measurement was used to build a feature matrix. NMF was used to decompose these features into two separate matrices, the basis, which describes the composition of each signature and the coefficient, which reports how prominent each signature is in each cell line and stem cell. The number of components parameter used for each decomposition was decided by running many NMF decompositions with increasing parameters, and choosing the number of components where the reconstruction error plateaued. Finally, we used hierarchical clustering on the coefficient matrices in order to cluster GBM cell lines and stem cells based on signature composition similarity (Fig. 2b, d, respectively).

### Reporting summary

Further information on research design is available in the Nature Research Reporting Summary linked to this article.

## Supplementary information

Supplementary Information

Description of additional supplementary items

Supplementary Data 1

Supplementary Data 2

Supplementary Data 3

Supplementary Data 4

Supplementary Data 5

Supplementary Data 6

Supplementary Data 7

Supplementary Data 8

Supplementary Data 9

Reporting Summary

## Data Availability

All relevant data are available from the authors upon request. The mass spectrometry proteomics data have been deposited to the ProteomeXchange Consortium via the PRIDE partner repository with the dataset identifier PXD014579^65^.
