# Supplementary material for: Cell-derived extracellular vesicles can be used as a biomarker reservoir for glioblastoma tumor subtyping
Source: Commun Biol. 2019 Aug 19;2:315. doi: 10.1038/s42003-019-0560-x (PMC6700082; doi:10.1038/s42003-019-0560-x)
Supplement: Supplementary file 1 — Supplementary Information [file 42003_2019_560_MOESM1_ESM.pdf]

Supplementary Figure 1

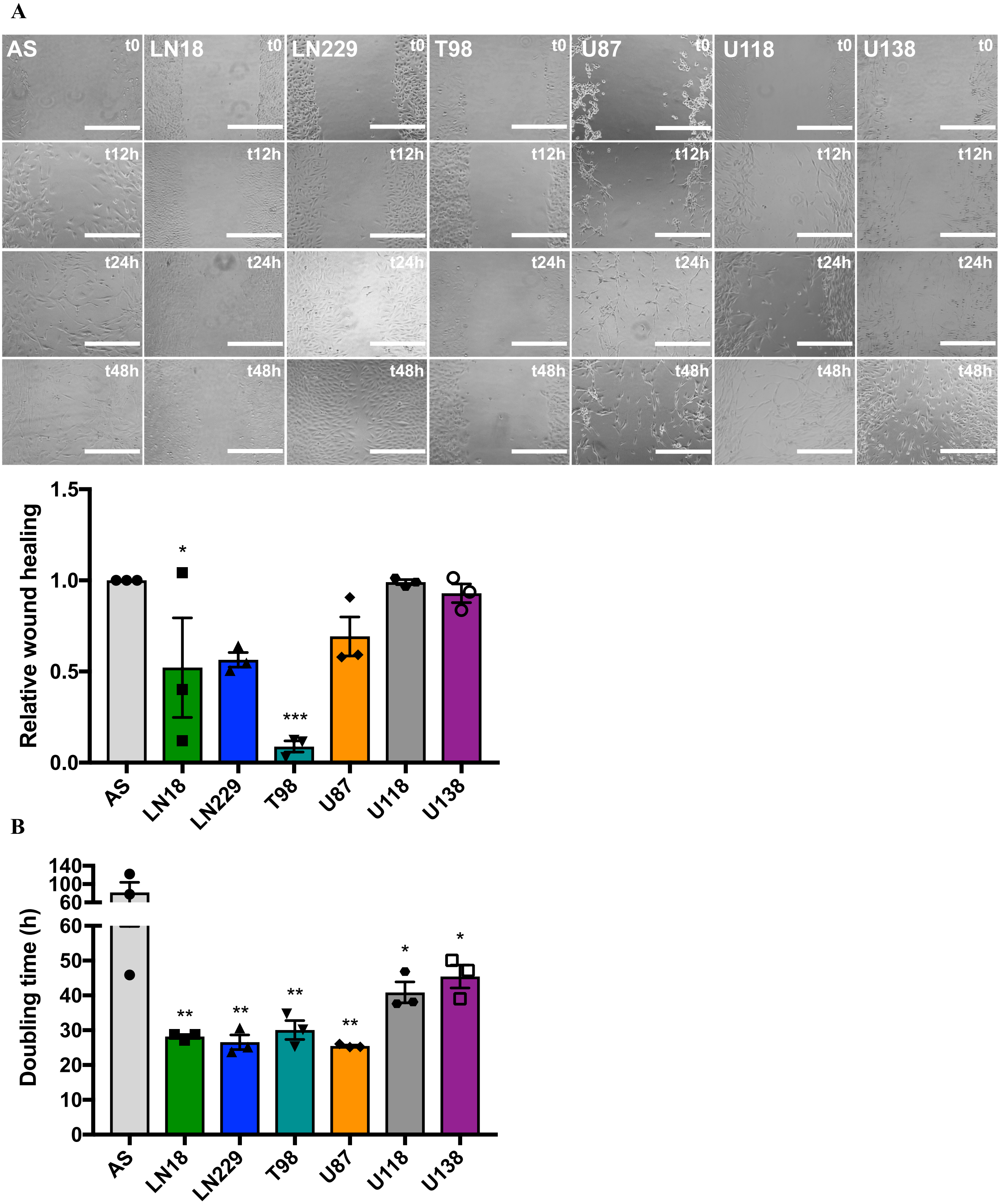

Supplementary Figure 1: Astrocytes and GBM cell lines present different *in vitro* wound healing, migration, proliferation and extracellular matrix invasion capabilities (A) Migration abilities of AS and GBM cell lines using wound healing assay. Cells were grown until confluence. A wound was formed in the cell layer and cells were allowed to grow and close the wound for 48h. Pictures were taken at time (t) 0, t12h, t24h and t48h. Scale bar=400µm. Size of the wound was then measured and wound healing accordingly calculated following normalization to original size at t0. **(B)** Cell doubling time of AS and GBM cell lines using a crystal violet assay. Cells were grown for 24h and 96h, then fixed and stained with crystal violet. Absorbance (590nm) was read at both time point and doubling time (h) accordingly calculated. The mean ± SEM of n=3 independent experiments is shown. *p<0.05, **p<0.01, ***p<0.001, **** p<0.0001(ordinary one-way ANOVA).

Supplementary Figure 2

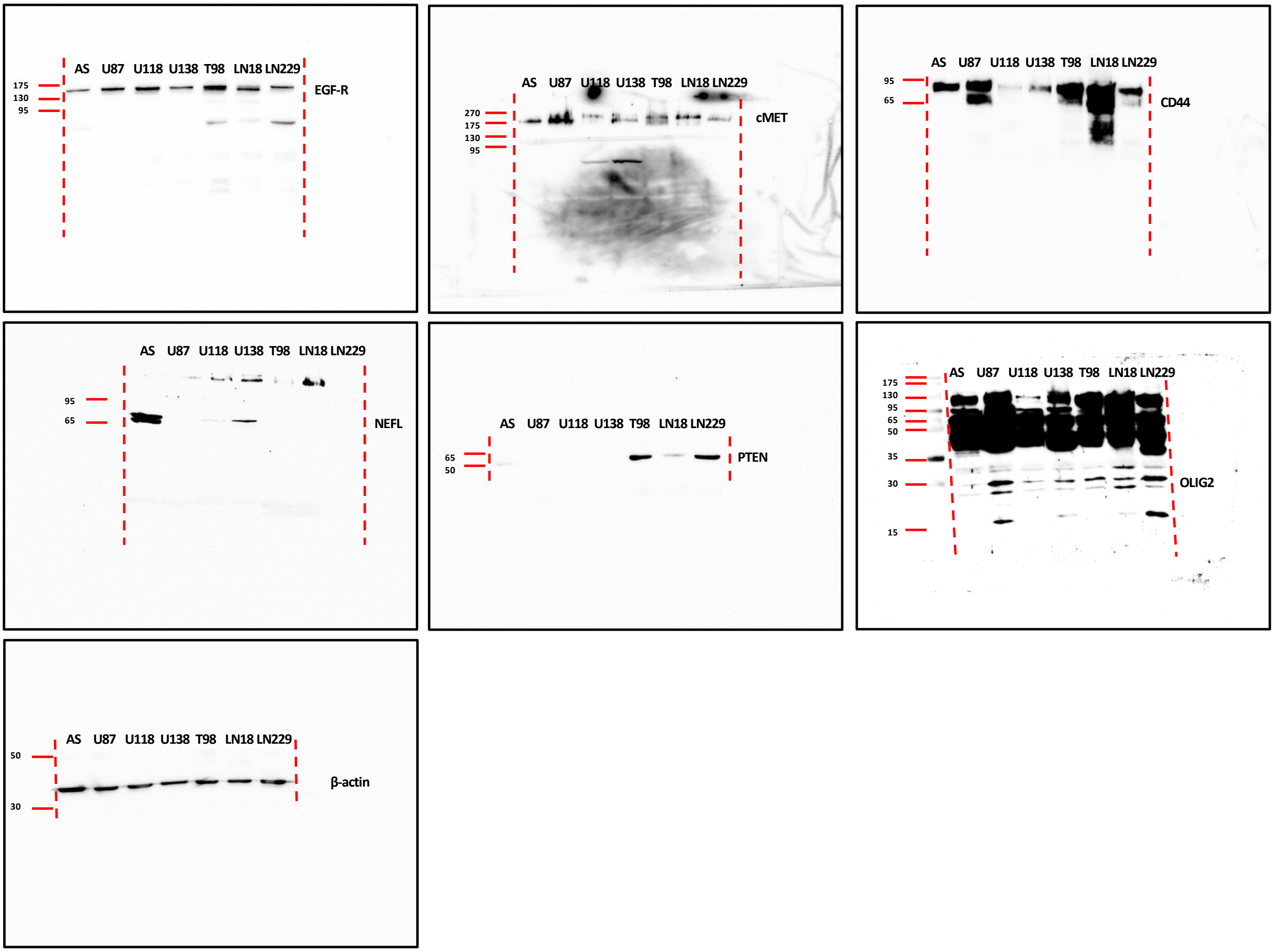

Supplementary Figure 2: Western blotting analysis of GBM subtype and aggressiveness marker expression in astrocytes and 6 different GBM cell lines. β-actin was used as an internal control. Uncropped images (cf **Figure 1E**).

Supplementary Figure 3

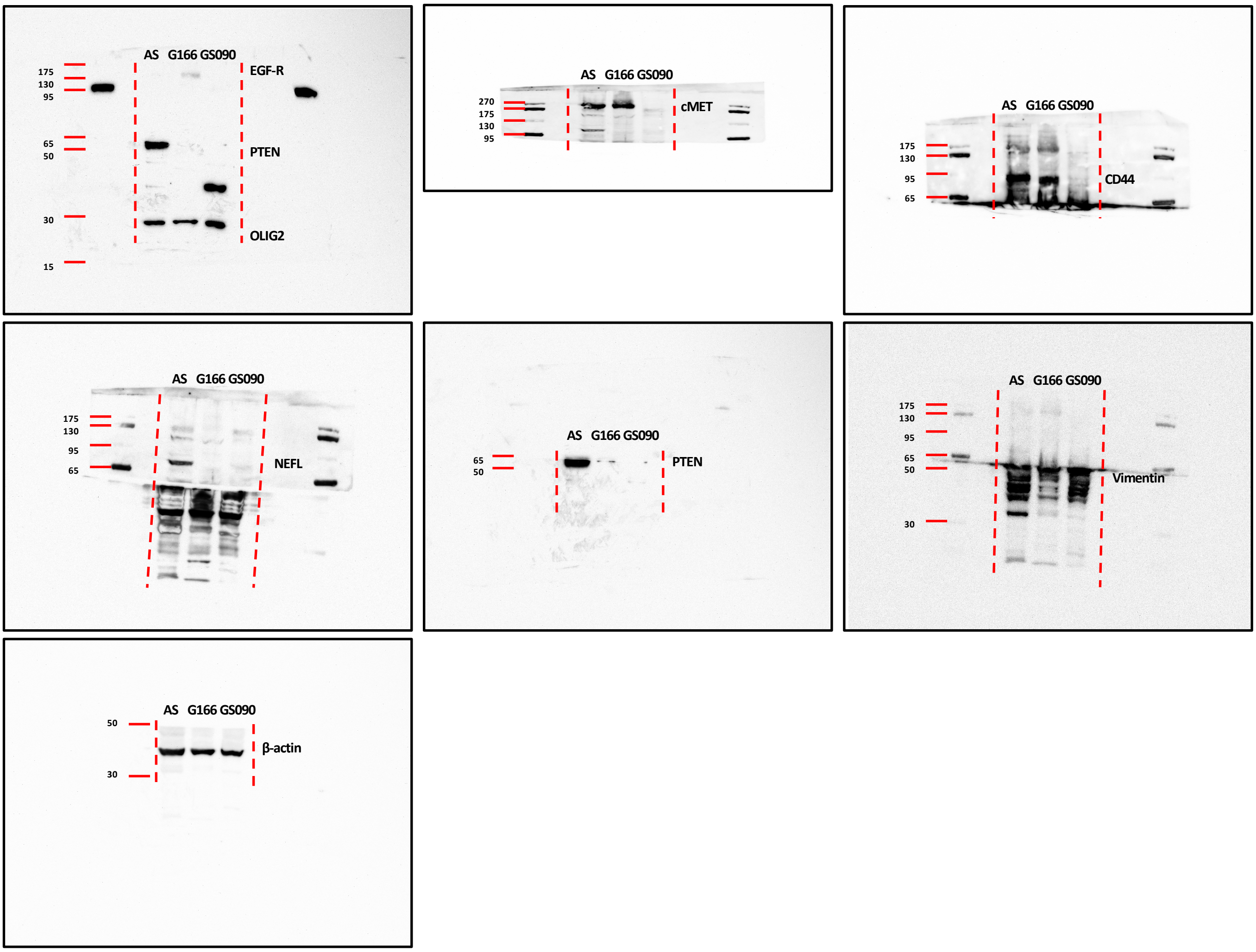

Supplementary Figure 3: Western blotting analysis of GBM subtype and aggressiveness marker expression in astrocytes and 2 different GBM patient-derived stem cells. β-actin was used as an internal control. Uncropped images (cf **Figure 1F**).

Supplementary Figure 4

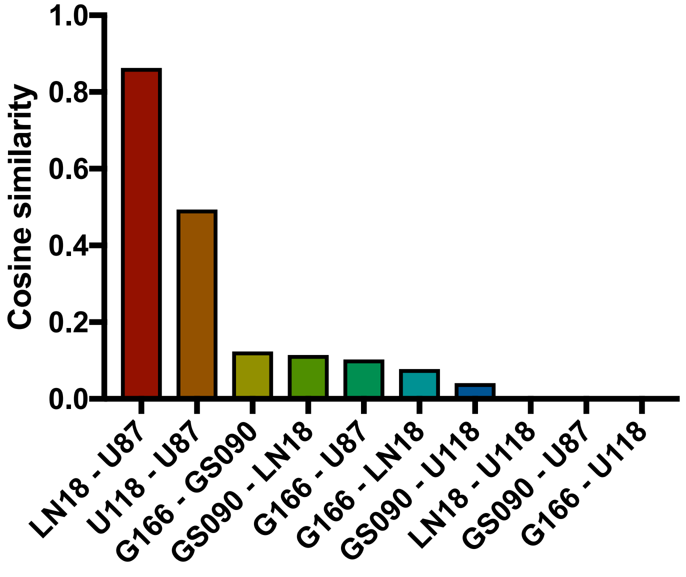

Supplementary Figure 4: Measurement of similarity between LN18, U87, U118, G166 and GS090 GBM cells. A cosine similarity assay was performed based on the 4 signature clustering data shown in **Figure 2D**.

Supplementary Figure 5

LN18

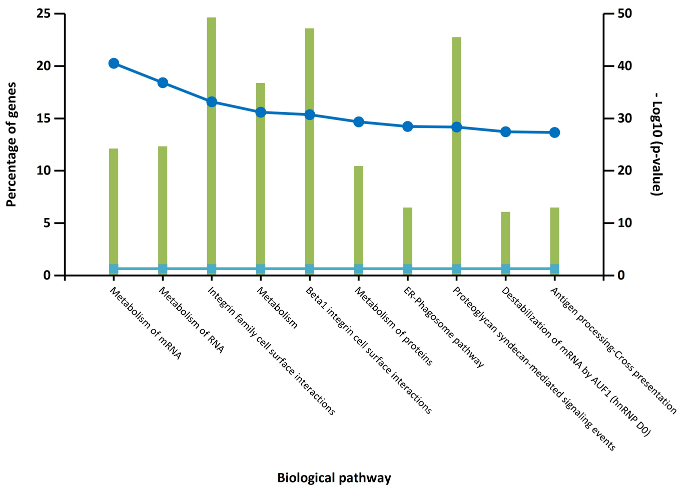

U87

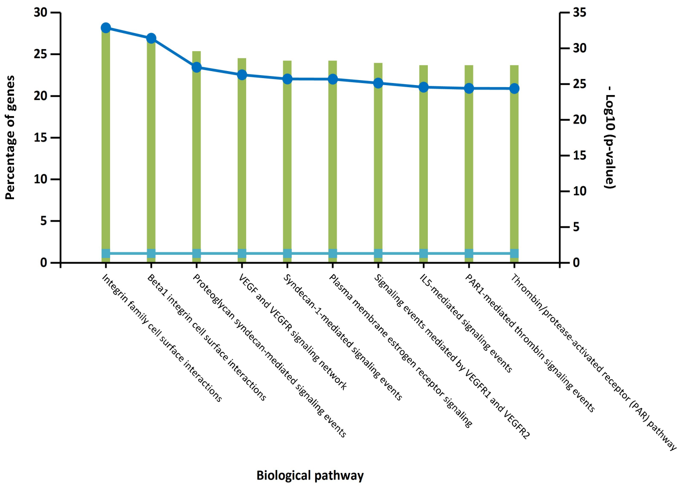

U118

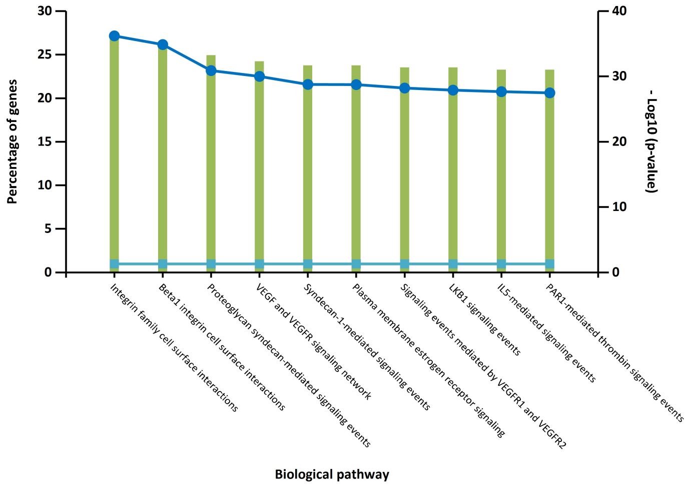

G166

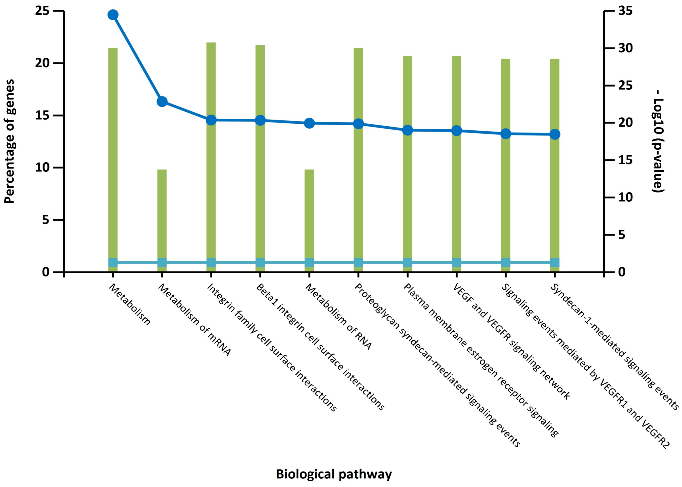

GS090

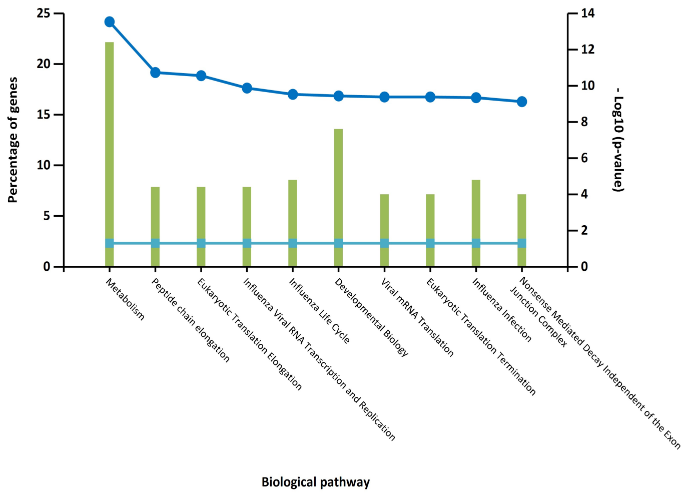

Supplementary Figure 5: Identification of biological pathways in the proteome of GBM cell-derived sEVs. Gene enrichment analysis for 'Biological pathway' was performed based on the MS hits identified for each GBM cell-derived sEVs

Supplementary Figure 6

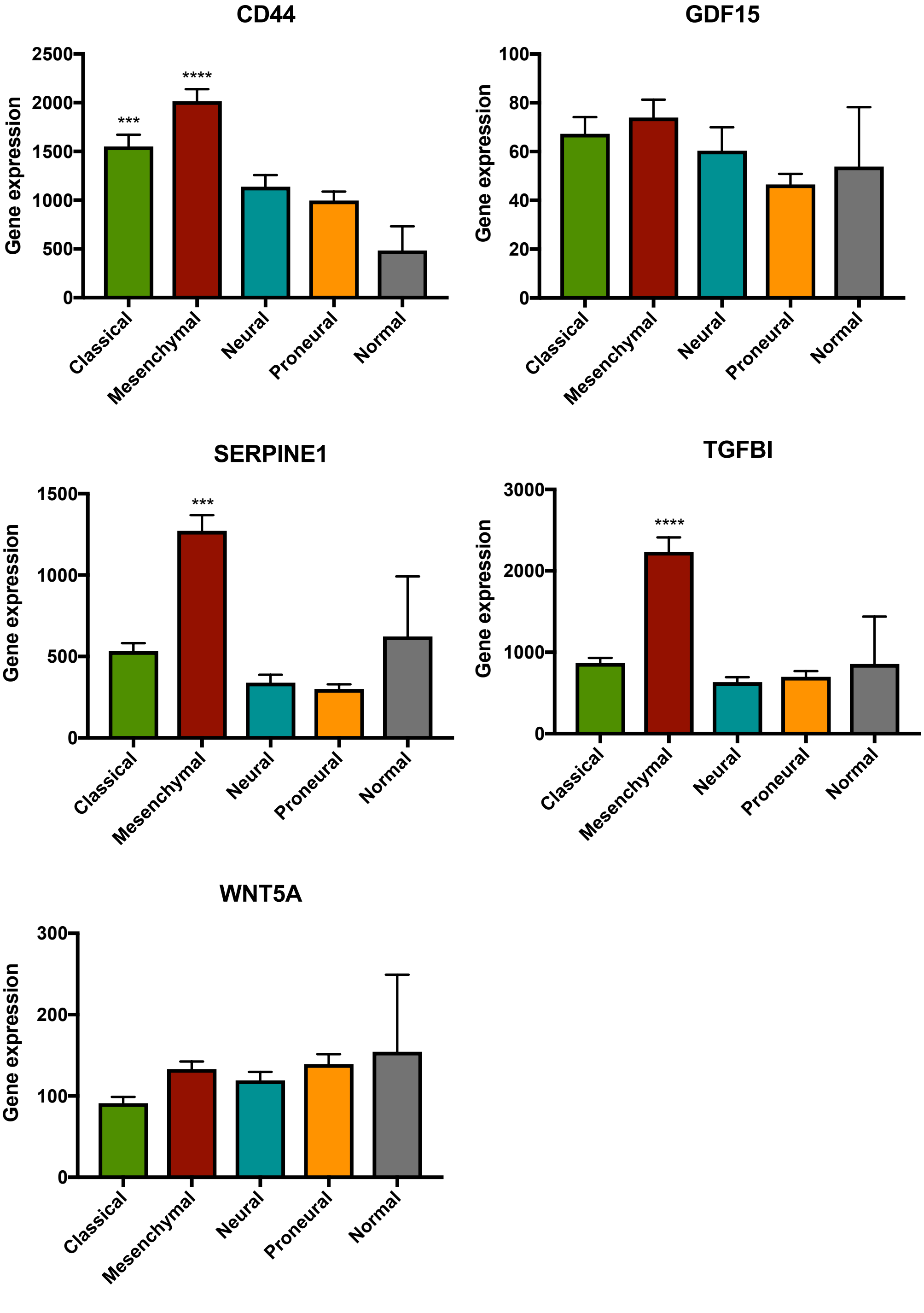

Supplementary Figure 6: Analysis of the gene expression of potential sEV-associated biomarkers for the GBM mesenchymal subtype. Potential biomarkers have been identified among the protein hits exclusively and commonly present in sEVs derived from LN18 and U87 GBM cells. Gene expression distribution of the specific hits among the different GBM subtypes has been obtained from The Cancer Genome Atlas (TCGA). *p<0.05, **p<0.01, ***p<0.01 (ordinary one-way ANOVA).

Supplementary Figure 7

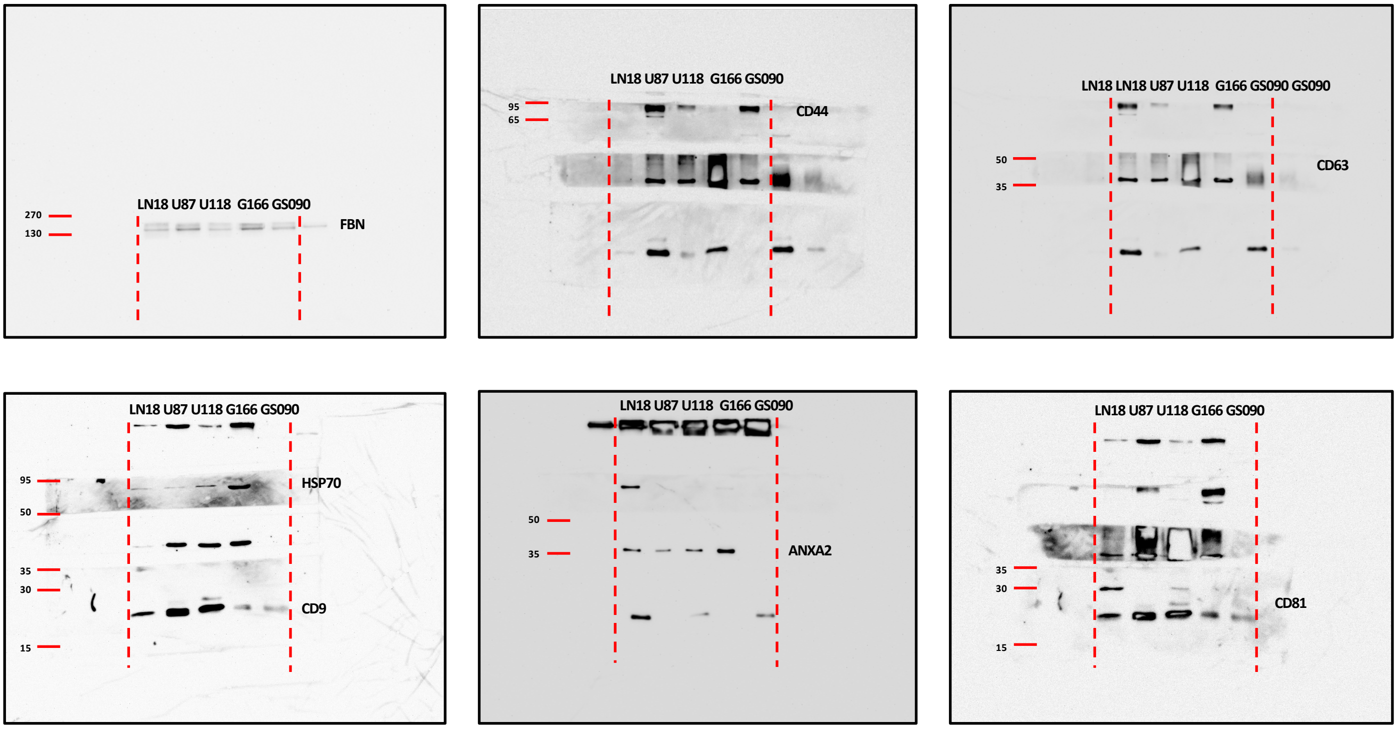

Supplementary Figure 7: Western blotting detection of fibronectin (FBN), CD44, CD63, HSP70, AnnexinA2 (ANXA2), CD9 and CD81 in GBM cell-derived sEVs. Uncropped images (cf **Figure 4F**).
