# Supplementary material for: Cell-derived extracellular vesicles can be used as a biomarker reservoir for glioblastoma tumor subtyping
Source: Commun Biol. 2019 Aug 19;2:315. doi: 10.1038/s42003-019-0560-x (PMC6700082; doi:10.1038/s42003-019-0560-x)
Supplement: Supplementary file 2 — Description of additional supplementary items [file 42003_2019_560_MOESM2_ESM.docx]

**Description of additional supplementary items**

*Source data underlying the graphs and charts presented in the main figures, as described in the title*

Supplementary Data1_Figure1_a-c – Figure 1: Astrocytes, GBM cell lines and GBM patient-derived stem cells present different *in vitro* invasion capabilities and specific subtype marker expression (a-c)*. –* Microsoft Excel file

Supplementary Data2_SupplementaryFigure1 – Supplementary Figure 1: Astrocytes and GBM cell lines present different *in vitro* wound healing, migration, proliferation and extracellular matrix invasion capabilities. – Microsoft Excel file

Supplementary Data3_Figure1_d – Figure 1: Astrocytes, GBM cell lines and GBM patient-derived stem cells present different *in vitro* invasion capabilities and specific subtype marker expression (d)*. –* Microsoft Excel file

Supplementary Data4_Figure1_g – Figure 1: Astrocytes, GBM cell lines and GBM patient-derived stem cells present different *in vitro* invasion capabilities and specific subtype marker expression (g)*. –* Microsoft Excel file

Supplementary Data5_Figure2 – Figure 2: Different groups of GBM cells can be defined based on invasiveness potential and marker expression data. – Microsoft Excel file

Supplementary Data6_SupplementaryFigure4 – Supplementary Figure 4: Measurement of similarity between LN18, U87, U118, G166 and GS090 GBM cells. A cosine similarity assay was performed based on the 4 signature clustering data shown in Figure 2D. – Microsoft Excel file

Supplementary Data7_Figure3 – Figure 3: sEV fractions produced by different GBM cell lines and patient-derived stem cells show variable concentrations and specific patterns of EV markers expression. – Microsoft Excel file

Supplementary Data8_Figure4_MS data – Figure 4: MS analysis reveals sEV proteomic content that mirrors GBM cell clustering signature and invasiveness *in vitro*. – Microsoft Excel file

Supplementary Data9_SupplementaryFigure6 – Supplementary Figure 6: Analysis of the gene expression of potential sEV-associated biomarkers for the GBM mesenchymal subtype. – Microsoft Excel file
