# Supplementary material for: Cell-derived extracellular vesicles can be used as a biomarker reservoir for glioblastoma tumor subtyping
Source: Commun Biol. 2019 Aug 19;2:315. doi: 10.1038/s42003-019-0560-x (PMC6700082; doi:10.1038/s42003-019-0560-x)
Supplement: Supplementary file 12 — Reporting Summary [file 42003_2019_560_MOESM12_ESM.pdf]

nature research

Corresponding author(s): Prof Georgios Giamas & Dr Thomas Simon

Last updated by author(s): Jul 9, 2019

Reporting Summary

Nature Research wishes to improve the reproducibility of the work that we publish. This form provides structure for consistency and transparency in reporting. For further information on Nature Research policies, see Authors & Referees and the Editorial Policy Checklist.

Statistics

For all statistical analyses, confirm that the following items are present in the figure legend, table legend, main text, or Methods section.

n/a	Confirmed	
☐	☒	The exact sample size (*n*) for each experimental group/condition, given as a discrete number and unit of measurement
☐	☒	A statement on whether measurements were taken from distinct samples or whether the same sample was measured repeatedly
☐	☒	The statistical test(s) used AND whether they are one- or two-sided<br>*Only common tests should be described solely by name; describe more complex techniques in the Methods section.*
☐	☒	A description of all covariates tested
☐	☒	A description of any assumptions or corrections, such as tests of normality and adjustment for multiple comparisons
☐	☒	A full description of the statistical parameters including central tendency (e.g. means) or other basic estimates (e.g. regression coefficient) AND variation (e.g. standard deviation) or associated estimates of uncertainty (e.g. confidence intervals)
☒	☐	For null hypothesis testing, the test statistic (e.g. *F*, *t*, *r*) with confidence intervals, effect sizes, degrees of freedom and *P* value noted<br>*Give P values as exact values whenever suitable.*
☒	☐	For Bayesian analysis, information on the choice of priors and Markov chain Monte Carlo settings
☒	☐	For hierarchical and complex designs, identification of the appropriate level for tests and full reporting of outcomes
☒	☐	Estimates of effect sizes (e.g. Cohen's *d*, Pearson's *r*), indicating how they were calculated

Our web collection on statistics for biologists contains articles on many of the points above.

Software and code

Policy information about availability of computer code

Data collection	BCA, Bradford, Crystal violet, CellTiter-Glo, CyQuant and ELISA assays were performed using a GloMax Explorer plate reader/software. Western blotting was performed using UVP Chemstudio instrument (Analytik Jena) and the Vision Works software. PCR was performed on a One-Step® Plus machine (Applied Biosystems), using the One-Step® Plus software. Nanoparticle Tracking Analysis was performed using a Nanosight© NS300 and the Nanosight© NTA 3.2 software (Malvern Instruments). Transmission Electron Microscopy samples were visualized using a JEOL JEM1400-Plus (120kV, LaB6) microscope and integrated software. Raw Mass Spectrometry data was searched using Maxquant (Max Planck Institute of Biochemistry).
Data analysis	Data analysis has been performed using Microsoft Excel (Version 14.6.5), GraphPad Prism 7 and FunRich (V3).

For manuscripts utilizing custom algorithms or software that are central to the research but not yet described in published literature, software must be made available to editors/reviewers. We strongly encourage code deposition in a community repository (e.g. GitHub). See the Nature Research guidelines for submitting code & software for further information.

Data

Policy information about availability of data

All manuscripts must include a data availability statement. This statement should provide the following information, where applicable:

- Accession codes, unique identifiers, or web links for publicly available datasets
- A list of figures that have associated raw data
- A description of any restrictions on data availability

All relevant data are available from the authors upon request. The mass spectrometry proteomics data have been deposited to the ProteomeXchange Consortium via the PRIDE partner repository with the dataset identifier PXD014579 (username: reviewer82463@ebi.ac.uk, password lpUv2vYV) .

Field-specific reporting

Please select the one below that is the best fit for your research. If you are not sure, read the appropriate sections before making your selection.

☒ Life sciences ☐ Behavioural & social sciences ☐ Ecological, evolutionary & environmental sciences

For a reference copy of the document with all sections, see nature.com/documents/nr-reporting-summary-flat.pdf

Life sciences study design

All studies must disclose on these points even when the disclosure is negative.

Sample size	Sample size was set to a minimum of 3 independent experiments (biological repeats) based on the magnitude and consistency of differences between cells/conditions.
Data exclusions	No data exclusion.
Replication	Experimental findings were reliably reproduced.
Randomization	Not applicable (no animal model involved in the present study).
Blinding	Not applicable (no animal model involved in the present study).

Reporting for specific materials, systems and methods

We require information from authors about some types of materials, experimental systems and methods used in many studies. Here, indicate whether each material, system or method listed is relevant to your study. If you are not sure if a list item applies to your research, read the appropriate section before selecting a response.

Materials & experimental systems

n/a	Involved in the study	
☐	☒	Antibodies
☐	☒	Eukaryotic cell lines
☒	☐	Palaeontology
☒	☐	Animals and other organisms
☒	☐	Human research participants
☒	☐	Clinical data

Methods

n/a	Involved in the study	
☒	☐	ChIP-seq
☒	☐	Flow cytometry
☒	☐	MRI-based neuroimaging

Antibodies

Antibodies used	Primary antibodies: Anti-AnnexinA2 (Genscript A01471, 1/1000 dilution), anti-β-Actin (Abcam ab6276, 1/5000 dilution), anti-CD-9 (System Biosciences EXOAB-CD9A-1, 1/10000 dilution), anti-CD44 (Cell Signaling #3570, 1/1000 dilution), anti-CD63 (System Biosciences EXOAB-CD63A-1, 1/10000 dilution), anti-CD81 (System Biosciences EXOAB-CD81A-1, 1/10000 dilution), anti-Fibronectin (Abcam ab2413, 1/1000 dilution), anti-HSP-70 (System Bisociences EXOAB-HSP70A-1,1/10000 dilution), anti-NEFL (Cell Signaling #2837, 1/1000 dilution), anti-OLIG2 (Genscript A01474, 1/1000 dilution) and anti-PTEN (Cell Signaling #9188, 1/1000 dilution). Secondary antibodies used: Polyclonal Goat Anti-Rabbit/Mouse Immunoglobulins/HRP (Dako P0447/8, 1/3000 dilution) antibodies and Anti-Rabbit Immunoglobulins/HRP (ExoAb antibody Kit, System Biosciences EXO-AB-HRP, 1/3000 dilution).
Validation	As described on the manufacturers' websites.

Eukaryotic cell lines

Policy information about cell lines

Cell line source(s)	ATCC
Authentication	As stated on the ATCC's website 'Cell lines from ATCC have been thoroughly tested and authenticated, so you can be certain of their identity.'
Mycoplasma contamination	All cell lines have been tested negative for mycoplasma contamination at the beginning of the study
Commonly misidentified lines (See ICLAC register)	

The ICLAC identifies U118 as a derivative of U138 as they appeared to share a common donor. Nevertheless, considering the GBM intra-tumoral heterogeneity, we decided to use both cell lines in the present study to compare them with each other and with the rest of the cells we used.
